# Comment on the “Diagnostic value of a computer- -assisted diagnosis system for the ultrasound features in thyroid nodules”

**DOI:** 10.20945/2359-4292-2024-0419

**Published:** 2025-02-24

**Authors:** Ahmet Bozer

**Affiliations:** 1 Department of Radiology, Izmir City Hospital, Izmir, Turkey

**DEAR EDITOR**,

I have carefully reviewed the article titled *“Diagnostic Value of a Computer-Assisted Diagnosis System for the Ultrasound Features in Thyroid Nodules”,* and I would like to commend the authors for their significant efforts in exploring the utility of the TUIAS (SW_TH01/II) CAD system (^1^). This research provides a valuable contribution to the growing body of evidence regarding the application of artificial intelligence in evaluating thyroid nodules. However, I would like to offer a few comments and suggestions for further consideration.

The authors have conducted a well-structured retrospective study that effectively evaluates the diagnostic accuracy of the CAD system in identifying key ultrasound features, such as aspect ratio, margin irregularity, and echogenicity. The high diagnostic performance of the CAD system in these parameters is noteworthy. The significant reduction in analysis time compared to manual evaluations by experienced sonographers underscores the potential clinical utility of this system, especially in busy healthcare settings where efficiency is crucial.

The study also highlights the CAD system’s ability to reduce inter-operator variability, a key issue in ultrasound diagnostics. The potential for this technology to assist in standardizing the assessment of thyroid nodules, especially in environments where experienced radiologists may not be available, is a notable strength of the study.

The findings show a high level of accuracy, sensitivity, and specificity across most of the features analyzed by the CAD system, which suggests that the system could help improve the consistency and accuracy of thyroid nodule evaluation. However, the study’s focus on static images limits its real-world applicability. In clinical practice, dynamic ultrasound features, such as blood flow and cervical lymph node involvement, are critical in determining the malignancy risk of thyroid nodules. Since these factors were not assessed in the study, the broader clinical implications of the system remain uncertain and call for further research.

One significant limitation is the study’s reliance on static ultrasound images, which restricts the evaluation to a single frame per nodule. In contrast, real-time ultrasound captures dynamic features, essential for a more comprehensive assessment of thyroid nodules. Moreover, the study does not account for critical factors, such as cervical lymph node evaluation and vascularity, which are often key in malignancy risk stratification. Further research should explore including these parameters in CAD systems to enhance diagnostic accuracy.

I should note that the retrospective and single-center nature of the study limits its generalizability. Larger, multi-center studies with prospective designs would provide more robust data and help validate the system’s performance in diverse clinical settings. Integrating advanced imaging techniques, such as contrast-enhanced ultrasound or elastography, could also further enhance the system’s ability to distinguish between benign and malignant nodules.
